# Combined tests with Xpert MTB/RIF assay with bronchoalveolar lavage fluid increasing the diagnostic performance of smear-negative pulmonary tuberculosis in Eastern China

**DOI:** 10.1017/S095026882000309X

**Published:** 2020-12-28

**Authors:** Qiao Liu, Ye Ji, Leonardo Martinez, Wei Lu, Xudong Shi, Jianming Wang, Yi Zeng

**Affiliations:** 1 Department of Chronic Communicable Disease, Center for Disease Control and Prevention of Jiangsu Province, Nanjing, China; 2 Department of Epidemiology, Center for Global Health, School of Public Health, Nanjing Medical University, Nanjing, China; 3 Division of Infectious Diseases and Geographic Medicine, School of Medicine, Stanford University, Stanford, USA; 4 Department of Epidemiology, Boston University, School of Public Health, Boston, Massachusetts, USA; 5 Department of Tuberculosis, Nanjing Public Health Medical Center, Nanjing Second Hospital, Nanjing Hospital Affiliated to Nanjing University of Traditional Chinese Medicine, Nanjing, China

**Keywords:** BALF, smear-negative, tuberculosis, Xpert MTB/RIF assay

## Abstract

Tuberculosis (TB) remains a global public health threat. Misdiagnosis and delayed therapy of sputum smear-negative TB can affect the treatment outcomes and promote pathogen transmission. The application of Xpert MTB/RIF assay in bronchoalveolar lavage fluid (BALF) has been recommended but needs clinical evidence. We carried out a prospective study in the Nanjing Public Health Medical Center from September 2018 to August 2019. Pulmonary tuberculosis (PTB) patients were enrolled in the study if they had negative results of sputum smear. We compared the performance of Xpert MTB/RIF assay in sputum and BALF using sputum culture as the reference. In addition to this, we applied parallel tests using sputum culture, sputum-based Xpert MTB/RIF assay and BALF-based Xpert MTB/RIF assay to jointly detect smear-negative PTB using clinical diagnosis as the reference. With mycobacterial culture as the reference standard, Xpert MTB/RIF of BALF showed a higher sensitivity (14/16, 87.5%), but a relatively lower specificity (57/92, 62.0%). Xpert MTB/RIF of sputum showed relatively lower sensitivity (6/10, 60.0%) and higher specificity (63/88, 71.6%). Compared with sputum culture, Xpert MTB /RIF assay reduced the median detection time of MTB from 30 to 0 days, which significantly shortened the diagnosis time of the smear-negative TB patients. Among the combined detections, the positive detection proportion was improved with significant differences comparing with sputum culture only, from 11.1% (10/90) to 46.7% (42/90) (*P* < 0.05). Our study showed Xpert MTB/RIF in BALF had a better performance in detecting MTB of smear-negative patients.

## Introduction

On a global scale, tuberculosis (TB) is one of the top 10 causes of death and the leading cause of a single infectious agent. Millions of people continue to suffer from TB every year. Globally, an estimated 10.0 million people fell ill with TB in 2018, most of whom were found in 30 high TB burden countries [1]. In 2014, the WHO put forward the End TB Strategy, which was shaped with a vision of making the world free of TB, with zero deaths, disease and suffering due to the disease [2].

Rapid detection of smear-negative TB is critical to improving health, reducing deaths and breaking the transmission of TB. Commonly used laboratory tests for TB are sputum smear microscopy examination and culture. Although culture is considered the reference standard, it is a time-consuming and complex operation, requiring a developed laboratory capacity and highly skilled staff [3]. The WHO recommends Xpert MTB/RIF assay (Cepheid Inc., Sunnyvale, CA, USA) for rapid detection for TB. Xpert MTB/RIF is an automated polymerase chain reaction test utilizing the GeneXpert platform [4, 5]. This fully automatic integration reaction does not require the pre-treatment process of the sample and has a strong ability to kill MTB, thus eliminating concerns about biosafety during testing [6]. Besides, Xpert MTB/RIF is faster (within 2 h) than previous diagnostic methods and can detect MTB complex and the resistance to rifampicin at the same time. The detection limit of Xpert MTB/RIF is 5 genomic copies of purified DNA in each reaction, or 131 colony forming units per ml of MTB [5], which is significantly lower than the sputum smear microscopy and mycobacterial culture [1].

Obtaining high-quality biological specimens for testing is key to reducing the diagnostic delay. Due to the number and/or insufficient quality of sputum samples, the detection rate of MTB might be low [7–11]. Bronchoalveolar lavage fluid (BALF) may be an alternative to sputum specimens to diagnose MTB, especially for patients who fail to provide adequate amounts or sufficient quality of sputum [12]. Xpert MTB/RIF assay of BALF samples could detect 33.9% of cases with negative MTB culture, and 48.7% of cases with negative acid-fast bacilli microscopy of childhood pulmonary TB (PTB) [13]. A parallel test carried out in Shanghai, China, has shown that the sensitivity was significantly higher in BALF than that in sputum samples of sputum smear-negative patients [14]. A prospective observational study in India found that among 31 Xpert MTB/RIF-positive cases, only nine of them were BALF-based culture-positive [15].

Previous studies have reported that BALF-based Xpert MTB/RIF assay had a significantly elevated detection proportion of MTB [7, 16, 17], but its diagnostic value in smear-negative patients remained unclear. Thus, we performed a prospective study on the diagnostic performance of a combined test with sputum culture, Xpert MTB/RIF in BALF or sputum from smear-negative PTB in Eastern China.

## Methods

### Study participants

First, we recruited 172 PTB patients in the Second Hospital of Nanjing during September 2018 and August 2019. Among these patients, 116 were sputum smear-negative cases and also consented to participate in the study. The inclusion criteria were: PTB patient, aged >18 years old, negative results from at least three sputum smears (morning sputum, night sputum and instant sputum) and willingness to undergo sputum culture and one or both of Xpert in sputum and BALF. In total, eight patients refused to perform the BALF test while other exclusions were due to age or sputum smear results. Patients were diagnosed according to the National Diagnostic Criteria for Pulmonary Tuberculosis in China (WS 288-2008) (Appendix file 1). Following the microscopy examination, sputum smear-negative patients underwent a series of clinical examinations for TB diagnosis, including chest X-ray examinations, sputum culture and Xpert MTB/RIF in sputum and BALF. If all three sputum smear results were negative, then these patients were classified as smear-negative patients. Smear-negative TB patients were further tested for sputum culture and Xpert to determine whether they were bacteriological-positive. Bacteriologically positive patients were defined as one or more positive (smear, culture or Xpert) test for MTB. Meanwhile, the bacteriologically negative patients were defined as negative smear, culture and Xpert tests for MTB.

### BALF collection

Three sputum samples (morning sputum, night sputum and instant sputum) were collected before treatment enrolment. BALF samples were obtained 7–14 days after treatment initiation. Bronchoscopy was performed by experienced clinical specialists in dedicated suites to collect BALF. First, in the lung segment to be lavaged, 1–2 ml of 2% lidocaine was injected through a biopsy hole to perform local anaesthesia. Flexible bronchoscopes (model BF-P40 and model BF-P60, Olympus Medical, Tokyo, Japan) were used to quickly inject 10 ml 37 °C sterile saline for 6–7 times. We used a vacuum suction device (50–100 mmHg) to pump back about 40 ml BALF for MTB detection.

### Mycobacterial culture

According to the viscosity of the specimen, 1–2 times volume of 4% sodium hydroxide (NaOH) was added to the specimen for 15 min. The inoculation should be completed within 20 min. The 4% NaOH was commonly used to digest samples which can reduce the chance of contamination. After inoculation on LJ solid medium (Besso Biotechnology Co., LTD, Wuhan, China), the culture was continued in a 37 °C incubator, and the growth of the bacterial colonies and its appearance and pigment production were observed.

### Xpert MTB/RIF assay procedures

The Xpert MTB/RIF assay was performed according to the manufacturer's instructions. Samples were mixed with the treatment solution containing NaOH and isopropanol at a ratio of 1:2 or 1:3 if the sputum contained pyocyte. After shaking, the mixture sample was stood upright at room temperature for 15 min, and then transferred to the single-used multi-chamber plastic reaction box. The box was placed in the GeneXpert^TM^ Dx module (Cepheid Inc.) for automatic detection.

### Statistical analysis

Data were entered with EpiData 3.1 (EpiData Association, Odense, Denmark) and analysed using Stata 15.0 (Stata Corp., College Station, TX, USA). We used the sensitivity, specificity, positive predictive value (PPV) and negative predictive value (NPV) together with 95% confidence intervals to estimate the diagnostic performance. The NPV and PPV refer to smear-negative TB patients and do not apply to smear-positive patients. We compared the time to detection of MTB by mycobacterial culture and Xpert MTB/RIF assay. We compared the performance of sputum-based Xpert MTB/RIF and BALF-based Xpert MTB/RIF using mycobacterial culture as the reference standard. By referring to the clinical diagnosis, we compared the detection proportions of combined tests. We have classified the combined tests into two separate classifications. ‘Combined tests A’ was classified as a combined testing of both sputum mycobacterial culture and sputum-based Xpert MTB/RIF. ‘Combined tests B’ was including combined testing of sputum mycobacterial culture, sputum-based Xpert MTB/RIF assay and BALF-based Xpert MTB/RIF assay. The *P* value of <0.05 was considered statistically significant.

### Ethics statement

This study was approved by the Ethics Committee of Jiangsu Provincial CDC. Personal information of patients did not appear in this study. Written informed consent was obtained from all eligible TB patients prior to study commencement.

## Results

### Demographic characteristics of subjects

**Fig. 1.**
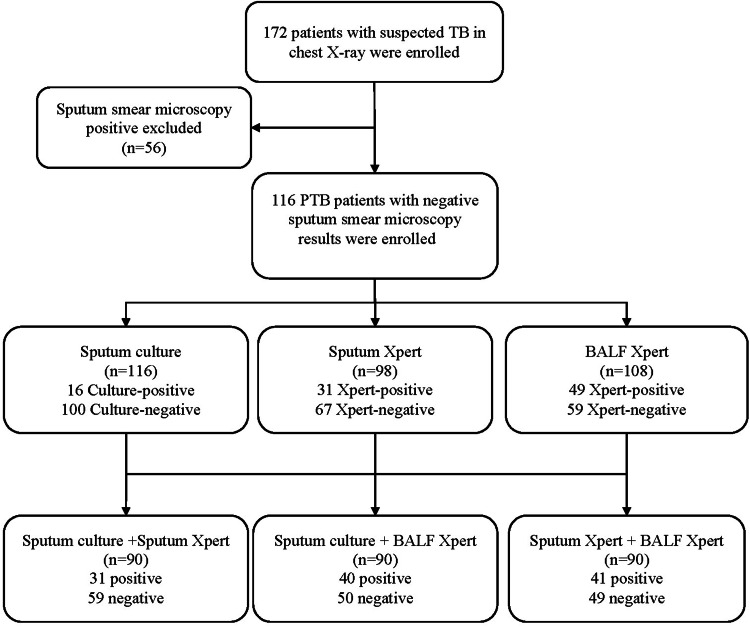
Flow chart of the study design. TB, tuberculosis; PTB, pulmonary tuberculosis; BALF, bronchoalveolar lavage fluid.

First, we recruited 172 PTB patients, and among them, 116 sputum smear-negative PTB cases were involved in the analysis (Fig. 1). There were 71 (61.2%) males and 45 (38.8%) females. A quarter (24.1%) of participants had a tobacco smoking history. Besides, nearly half of the subjects (49.1%) were bacteriological-positive. Both BMI (*P* < 0.0001) and tobacco smoking (*P* = 0.012) were statistically different between bacteriological-negative and positive groups (Table 1). Among the 116 participants, 26 only underwent one of the Xpert MTB/RIF assays in BALF and sputum samples. Specifically, there were 18 patients who only underwent BALF-based Xpert MTB/RIF and eight patients only underwent sputum-based Xpert MTB/RIF.

**Table 1.** Demographic characteristics of 116 tuberculosis patients

Variables	All (*N*, %)	Bacteriological negative (*N*, %)	Bacteriological positive (*N*, %)	*χ*^2^	*P* value
Gender				3.471	0.062
Male	71 (61.2)	41 (69.5)	30 (52.6)		
Female	45 (38.8)	18 (30.5)	27 (47.4)		
Age				7.654	0.054
≤25	31 (26.7)	13 (22.0)	18 (31.6)		
26–39	29 (25.0)	13 (22.0)	16 (28.1)		
40–55	29 (25.0)	13 (22.0)	16 (28.1)		
＞55	27 (23.3)	29 (33.9)	7 (12.3)		
BMI				16.445	<0.0001
<18.5	17 (14.7)	2 (3.4)	15 (26.3)		
≥18.5 to <24	72 (62.1)	38 (64.4)	34 (59.6)		
≥24 to <28	23 (19.8)	15 (25.4)	8 (14.0)		
≥28	4 (3.4)	4 (6.8)	0 (0.0)		
Smoking status				6.247	0.012
Never smoked	88 (75.9)	30 (66.1)	49 (86.0)		
Ever smoked	28 (24.1)	20 (33.9)	8 (14.0)		

BMI, body mass index.

### Performance of Xpert MTB/RIF assay using mycobacterial culture as the reference standard

The Standards for Reporting Diagnostic Accuracy Studies (STARD) table has been fully completed for evaluation and can be found in Appendix Table 1. As shown in Table 2, comparing sputum-based Xpert MTB/RIF, Xpert MTB/RIF in BALF had a higher sensitivity (87.5%), but a relatively lower specificity (62.0%). Xpert MTB/RIF in sputum showed lower sensitivity (60.0%) and higher specificity (71.6%). The NPV was 94.0% (85.4–98.3%) for sputum and 96.6% (88.3–99.6%) for BALF samples. Besides, BALF-based Xpert MTB/RIF assay detected 35 (38.04%) positive results out of 92 culture-negative cases, and sputum-based Xpert MTB/RIF assay detected 25 (28.41%) out of 88 of culture-negative cases.

**Table 2.** Performance of Xpert MTB/RIF assay using mycobacterial culture as the reference standard

		Positive	Negative	Sensitivity	Specificity	Accuracy	PPV	NPV
SX	Positive	6	25	60.0% (6/10) (26.2–87.8%)	71.6% (63/88) (61.0–80.7%)	70.4% (69/98) (60.3–79.2%)	19.4% (6/31) (7.5–37.5%)	94.0% (63/67) (85.4–98.3%)
	Negative	4	63					
BX	Positive	14	35	87.5% (14/16) (61.7–98.4%)	62.0% (57/92) (51.2–71.9%)	65.7% (71/108) (56.0–74.6%)	28.6% (14/49) (16.6–43.3%)	96.6% (57/59) (88.3–99.6%)
	Negative	2	57					

SX, sputum Xpert; BX, BALF Xpert; PPV, positive predictive value; NPV, negative predictive value.

### Comparison of Xpert MTB/RIF assay and mycobacterial culture on time to MTB detection

**Fig. 2.**
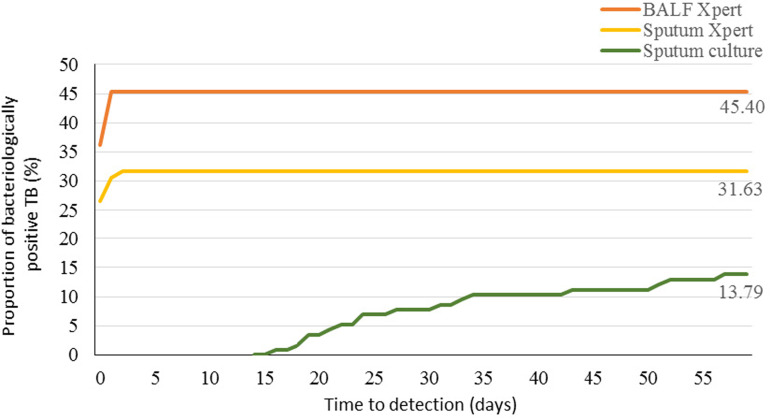
Comparison of Xpert MTB/RIF assay and mycobacterial culture on time to MTB detection. Percentages are the maximum proportion of MTB detected by every method. TB, tuberculosis; BALF, bronchoalveolar lavage fluid.

The time-specific proportions of MTB detected by Xpert MTB/RIF assay (sputum and BALF) and mycobacterial culture (sputum) were shown in Figure 2. The median detection time was 0 day for Xpert MTB/RIF assay (sputum or BALF), while it took about 27 days (ranged 21–40 days) for mycobacterial culture (sputum).

### Diagnostic performance of combined detection methods

**Fig. 3.**
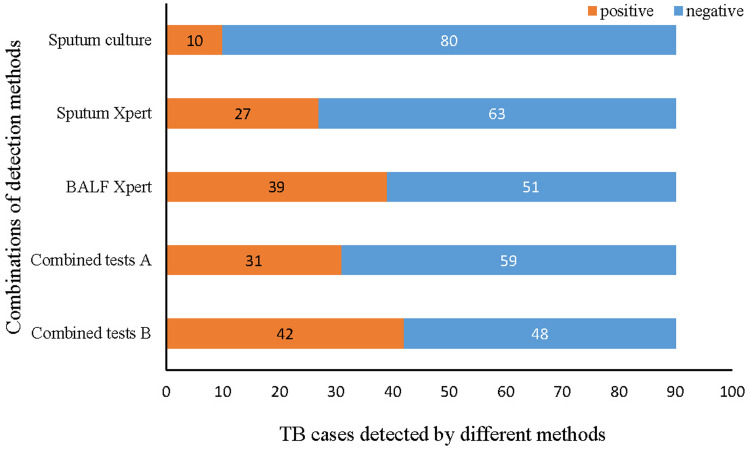
Sensitivity comparison of single tests and combined detections. TB, tuberculosis; SC, sputum culture; SX, sputum Xpert; BX, BALF Xpert.

We further estimated the performance of the combination of sputum mycobacterial culture, sputum-based Xpert MTB/RIF assay and BALF-based Xpert MTB/RIF assay. Of 90 patients with the results of three types of tests, the positive detection proportion was 43.3% (39/90) for BALF-based Xpert MTB/RIF assay, followed by sputum-based Xpert MTB/RIF assay (30.0%, 27/90) and sputum mycobacterial culture (11.1%, 10/90). The positive detection proportions of Xpert MTB/RIF assay of sputum and BALF were improved with significant differences (*P* = 0.002, *P* < 0.001) comparing with sputum mycobacterial culture. ‘Combined tests B’ demonstrated a higher positive detection proportion 46.7% (42/90) compared to ‘Combined tests A’, however this difference was not statistically significant (*P* > 0.05) (Fig. 3).

## Discussion

Our study demonstrated that Xpert MTB/RIF assay had a better performance than sputum mycobacterial culture in smear-negative PTB. Besides, combined detection tests had a higher sensitivity than the single one.

Despite the increase in TB notifications, there is still a significant gap between the number of new cases reported (7 million) and the estimated 10 million cases (ranging from 9 to 11.1 million) in 2018 [1]. This gap is largely attributed to a combination of underreporting of detected cases and underdiagnosis [18]. A large number of researches reported that many countries had made more efforts to improve the diagnosis of smear-negative TB and reduce the gap between morbidity and notification. The proportion of bacteriologically confirmed notified cases needs to be monitored to ensure that smear-negative TB patients are properly diagnosed and start timely and effective treatment options. The goal should be to increase the percentage of bacteriologically confirmed cases by expanding the use of recommended diagnostic methods that are more sensitive than mycobacterial culture, such as Xpert MTB/RIF assay. In our study, Xpert MTB/RIF assay of sputum and BALF samples both showed high sensitivity with clinical diagnosis as the reference standard. As 27 studies with almost 10 000 participants reported, the pooled sensitivities of Xpert for PTB were 98% in those who were positive by sputum smear microscopy but only 67% in those who were negative by sputum smear microscopy [19]. The sensitivity of sputum smear is 67.4%, which is consistent with other researches reporting sensitivities of smear microscopy ranging from 61.8% to 70% [20, 21].

Due to any deficiency in the key steps, the quality of the sputum collected by the patient may differ greatly, including the medical staff's instructions to the patient to understand the correct collection of sputum and the laboratory's assessment of sputum quality [8]. A prospective multicentre study in Switzerland suggested that if both on-the-spot and early-morning sputum samples were smear-negative, the diagnostic yield was increased by bronchoscopy and, to a lesser extent, by two samples of induced sputum [22]. Besides, Xpert MTB/RIF assay had a better performance of MTB detection in BALF [14]. In our study, the detection proportion of MTB by the sputum collected of the patient was lower than that of BALF.

At present, almost all single tests including smear, culture, molecular and immunological samples have defects of varying degrees clinically. A study conducted in Mozambique which combined Xpert MTB/RIF and LAM for MTB detection in HIV-positive individuals did increase case finding, and shorten time to treatment [23]. In our study, compared with sputum culture, Xpert MTB /RIF assay reduced the median detection time of MTB from 30 to 0 day, which greatly shortened the diagnosis time of the smear-negative TB patients. Previously published meta-analysis literature has demonstrated that certain methods, such as culture and nucleic acid amplification tests, were relatively reliable tests for diagnosing PTB [24–26]. In our study, the detection proportion of Xpert MTB/RIF assay combined with culture did increase. In addition, the combined detection results of BALF presented higher diagnostic values than sputum samples. Both of them proved that combined detection can further increase the diagnostic sensitivity for PTB.

Accurate and rapid detection of TB, including smear-negative TB and drug-resistant TB, is essential for improving patient outcomes (increasing cure rates and reducing mortality, increasing drug resistance, treatment failure and relapse) and reducing TB transmission. In 2018, 55% of PTB were bacteriologically confirmed, a slight decrease from 56% in 2017. In high-income countries that have access to the most sensitive diagnostic tests, about 80% of pulmonary TB cases are bacteriologically confirmed, China below 40% [1]. Most of the clinical features of TB, as well as chest radiology associated with TB, have low specificity, which can lead to misdiagnosis and result in unnecessary TB treatment. Although combined tests in our study increased the detection to 45.3%, there is still a way to go. In addition, strategies on the basis of BALF collection with Xpert MTB/RIF are concerned high cost, which could not meet cost-effectiveness in underdeveloped areas. However, collection of BALF is an invasive procedure that requires professional clinical training. This is only recommended for suspected PTB patients in regions with a high frequency of TB [27]. In the future, tests with higher sensitivity such as Xpert Ultra should be used for the diagnosis of smear-negative TB patients.

This study has several obvious limitations. First, it was only conducted in an infectious disease specialist hospital and may weaken the validity of the research findings. Therefore, in other cases, further evaluation is needed to clarify the incremental value of BALF in the diagnosis of TB. Second, although mycobacterial culture is still a reliable reference standard for confirming the presence of MTB in clinical specimens, its low positive proportion may affect the outcome of other assays. Third, we did not exclude patients who did not participate in all tests, and there may be selection bias. Lastly, the low sample size is also a limitation of this study and future large cohorts are needed to confirm these results. Nonetheless, this study provides an alternative to improving the bacteriological diagnosis of TB.

In summary, our study showed that detection tests based on Xpert MTB/RIF in BALF had a better performance in detecting MTB of smear-negative patients than conventional tests. Further study may find out whether higher costs can be offset by higher accuracy, shorter time and avoidable overtreatment for patients with suspected TB.

## Data Availability

The findings in this study do not rely on any data, code or other resources.

## Supplementary material

For supplementary material accompanying this paper visit https://doi.org/10.1017/S095026882000309X. click here to view supplementary material
