# Supplementary material for: Combined tests with Xpert MTB/RIF assay with bronchoalveolar lavage fluid increasing the diagnostic performance of smear-negative pulmonary tuberculosis in Eastern China
Source: Epidemiol Infect. 2020 Dec 28;149:e5. doi: 10.1017/S095026882000309X (PMC8057514; doi:10.1017/S095026882000309X)
Supplement: Supplementary file 1 [file S095026882000309Xsup.zip › S95026882000309Xsup001.docx]

# Appendix File. Clinical diagnostic criteria of TB-patients according to the national diagnostic criteria (WS 288-2008):

1. Bacteriologically confirmed TB pulmonary tuberculosis:

- Acid fast bacilli positive with 2 sputum samples in smear microscopy tests; or
- Acid fast bacilli positive with 1 sputum samples in smear microscopy tests, and active pulmonary tuberculosis by radiological examination; or
- Acid fast bacilli positive with 1 sputum samples in smear microscopy tests, and mycobacterium tuberculosis positive in 1 sputum culture; or
- Negative in sputum smear microscopy tests but positive in 1 sputum culture, and active pulmonary tuberculosis in radiological examination.

1. Not bacteriologically confirmed pulmonary tuberculosis:

- Negative in sputum smear microscopy tests and sputum culture, and radiological examination of active pulmonary tuberculosis with suspicious symptoms such as cough, expectoration, and hemoptysis; or
- Negative in sputum smear microscopy tests and sputum culture, and radiological examination of active pulmonary tuberculosis with strong positive result in TST (TB-PPD test); or
- Negative in sputum smear microscopy tests and sputum culture, and Radiological examination of active pulmonary tuberculosis with positive result in anti-tuberculosis antibody test; or
- Negative in sputum smear microscopy tests and sputum culture, and radiological examination of active pulmonary tuberculosis with tuberculosis positive in pathological examination of the extra-pulmonary lesions; or
- Negative in sputum smear microscopy tests and sputum culture, and suspected lung pulmonary tuberculosis cases determined by diagnostic therapy or follow-up observation with other possible causes of lung diseases being ruled out.
